# Retraction: Integrin-Mediated Signaling Induced by Simian Virus 40 Leads to Transient Uncoupling of Cortical Actin and the Plasma Membrane

**DOI:** 10.1371/journal.pone.0317529

**Published:** 2025-01-09

**Authors:** The PLOS ONE Editors

After this article [1] was published, concerns were raised regarding the results presented in Figs 2, 3, 5, and S5

Specifically:

- There appear to be vertical discontinuities in the background of the following blots suggestive of splicing:
  ○ Fig 2D VP1 and tubulin
  ○ Fig 2E VP1 and tubulin
  ○ Fig 2G VP1 and tubulin
  ○ Fig 3A p-AKT(Ser473)
  ○ Fig 3D p-AKT(Ser473) and tubulin
  ○ Fig 3F p-AKT(Ser473) and tubulin
  ○ Fig 5D RhoA-GTP
  ○ Fig S5E p-ERM (Thr567), p-Akt (Ser473), and tubulin
- Fig 2D upper and lower panels appear similar
- In Fig 2E tubulin blot, lane 5 and lane 6 appear similar

With regards to the western blot vertical discontinuities, the corresponding author stated that this may have originated from rearrangement of non-adjacent lanes on the same blot or film, but stated they did not observe all of the listed concerns. With regards to the similarities of the upper and lower panels of Fig 2D, the corresponding author indicated this was an erroneous duplication of an identical panel and should not have been included in the figure. The corresponding author stated that the original data are no longer available due to the age of the experiments. In the absence of these data the above concerns cannot be resolved and the *PLOS ONE* Editors retract this article.

All authors agreed with the retraction and apologize for the issues with the published article.

## References

[1] Stergiou L, Bauer M, Mair W, Bausch-Fluck D, Drayman N, Wollscheid B, et al. (2013) Integrin-Mediated Signaling Induced by Simian Virus 40 Leads to Transient Uncoupling of Cortical Actin and the Plasma Membrane. PLoS ONE 8(2): e55799. 10.1371/journal.pone.0055799 23409046 PMC3567119

